# Comparison between daily and on‐demand PrEP (pre‐exposure prophylaxis) regimen in covering condomless anal intercourse for men who have sex with men in Hong Kong: A randomized, controlled, open‐label, crossover trial

**DOI:** 10.1002/jia2.25795

**Published:** 2021-09-02

**Authors:** Tsz Ho Kwan, Grace Chung Yan Lui, Teddy Tai Ning Lam, Krystal Chi Kei Lee, Ngai Sze Wong, Denise Pui Chung Chan, Shui Shan Lee

**Affiliations:** ^1^ Stanley Ho Centre for Emerging Infectious Diseases The Chinese University of Hong Kong Shatin Hong Kong; ^2^ Department of Medicine and Therapeutics The Chinese University of Hong Kong Shatin Hong Kong; ^3^ School of Pharmacy The Chinese University of Hong Kong Shatin Hong Kong; ^4^ Department of Psychiatry Queen Mary Hospital Pokfulam Hong Kong

**Keywords:** Asia, clinical trials, crossover design, HIV prevention trials, men who have sex with men, PrEP

## Abstract

**Introduction:**

Both daily and on‐demand regimens have been proven effective for pre‐exposure prophylaxis (PrEP) against HIV in men who have sex with men (MSM). We aimed to compare the two regimens on their coverage of condomless anal intercourse (CLAI) in MSM.

**Methods:**

A randomized, controlled, open‐label, crossover trial was conducted in a teaching hospital in Hong Kong. Participants were sexually active HIV‐negative MSM aged 18 years or above with normal renal function and without chronic hepatitis B infection. Oral tenofovir disoproxil fumarate 300 mg/emtricitabine 200 mg (TDF/FTC) tablets were prescribed for PrEP. After a 2‐week lead‐in with daily TDF/FTC for treatment‐naïve MSM for tolerance assessment, participants were randomly assigned in a 1:1 ratio with a block size of four to either daily‐first or on‐demand‐first arm based on the IPERGAY study, for receiving PrEP for 16 weeks, then crossed‐over to the alternative regimen for another 16 weeks. The primary outcome was the proportion of days with PrEP‐covered CLAI by intention‐to‐treat analysis. The trial is registered with the CCRB Clinical Trials Registry, CUHK, CUHK_CCRB00606, and is closed to accrual.

**Results:**

Between 25 August 2018 and 23 March 2019, 119 eligible participants were assigned to daily‐first arm (*n* = 59) and on‐demand‐first arm (*n* = 60) with an 87% overall completion rate (*n* = 103). With 96% and 54% of days on PrEP during daily and on‐demand periods, respectively, the proportion of days with PrEP‐covered CLAI between two arms were not statistically different (92% vs. 92%, *p* = 0.93). About half (47%) were diagnosed with at least one episode of incident sexually transmitted infection. Mild and time‐limited adverse events, including diarrhoea, headache, nausea and dizziness, were reported in 37 (31%) and 10 (8%) during the daily and on‐demand periods, respectively. At the end of the study, a similar proportion favoured daily or on‐demand regimen.

**Conclusions:**

High prevention‐effective adherence, as reflected from the coverage of CLAI, was achievable by either daily or on‐demand PrEP among MSM, albeit a higher number of tablets taken for daily PrEP. As both regimens were well accepted, a flexible approach adopting either or both regimens with possible switching is warranted in order to suit individual health needs.

## 1 INTRODUCTION

Pre‐exposure prophylaxis (PrEP) with co‐formulated tenofovir disoproxil fumarate and emtricitabine (TDF/FTC) is a proven form of biomedical HIV prevention. Following the demonstration of its effectiveness in iPrEx, a placebo‐controlled trial [1], multiple studies with similar efficacy results have been published, as concluded in a review [2]. The World Health Organization and Centers for Disease Control and Prevention have formulated guidelines on the use of PrEP for people with substantial infection risk, notably men who have sex with men (MSM) [3, 4]. While daily PrEP is the standard regimen, event‐driven or on‐demand PrEP has also been shown to reduce the relative risk of HIV transmission in MSM, in a randomized controlled trial [5] and an observational study [6], of the ANRS IPERGAY Study Group. The efficacy of either on‐demand or daily PrEP was high in separate studies, but direct comparison between the two regimens as regards to their HIV prevention effects was limited. Previous studies showed that age and risk profile were associated with one's choice of PrEP regimen [7], which subsequently affected the sexually transmitted infection (STI) incidence during PrEP use [8]. In open‐label trials, breakthrough HIV infection was similarly uncommon for both daily and on‐demand PrEP [7, 8, 9, 10]. In Hong Kong, there have not been any government‐funded PrEP programmes and only patented TDF/FTC was available through prescriptions, the cost of which was well beyond the local MSM's affordability [11].

Despite the use of identical antiretrovirals, on‐demand and daily PrEP each requires very different forms of life adjustment. Studies have revealed that on‐demand PrEP could result in less protective coverage of condomless sex events [12, 13], which might imply lower effectiveness in the long run. If allowed to choose, the proportion of MSM who indicated preference of on‐demand versus daily PrEP varied between study cohorts from less than half [14, 15], about half [7], to three‐quarters [8, 9]. Preference for on‐demand PrEP was higher for MSM accessing TDF/FTC from informal sources [16]. Most published studies evaluating the two forms of PrEP almost exclusively involved MSM separately put on daily or on‐demand PrEP, while limited literature exists as regards to regimen switch, a phenomenon not uncommon in real‐life settings [9], which might be associated with changing relationships and risk patterns [15]. To address these research gaps while controlling for the inter‐personal variability of risk and behavioural factors, we conducted a randomized crossover trial to assess the impacts of the two regimens in the same cohort of MSM.

## 2 METHODS

### 2.1 Study design

This was a randomized controlled open‐label crossover trial comparing MSM's prevention‐effective adherence to daily and on‐demand PrEP, through examining the coverage of days with condomless anal intercourse (CLAI). The study was conducted in a teaching hospital in Hong Kong. A data and safety monitoring committee was in place for overseeing the study. The protocol was approved by the Joint Chinese University of Hong Kong – New Territories East Cluster Clinical Research Ethics Committee (Reference number: 2016.719). The clinical trial was approved by the Department of Health, Hong Kong (Certificate number 101143) and was registered in the Centre for Clinical Research and Biostatistics Clinical Trials Registry, The Chinese University of Hong Kong (Trial number: CUHK_CCRB00606) and Chinese Clinical Trial Registry (Registration number: ChiCTR1800016100).

### 2.2 Participants

Eligible participants were MSM, aged 18 years or above, who had had CLAI with men in the preceding 6 months, inclined to have CLAI in the coming 6 months, were HIV‐negative, not hepatitis B carriers, had a creatinine clearance of at least 60 ml/min and occurrence of at least one behavioural risk in the past 6 months, including chemsex engagement, STI diagnosis, had multiple sex partners and had people living with HIV (PLHIV) as sex partners regardless of their viral load status [11]. Participants were ineligible if they were unable to communicate in either Chinese or English, were not normally residing in Hong Kong, or had any form of mental illnesses. A sample size of 88 could give an 80% power to detect a 15% non‐inferiority margin of PrEP coverage of CLAI‐days with a 5% standard deviation at a one‐sided 2.5% confidence interval. To account for potential subject attrition, the target sample size was set at 120. Advertisements were placed in an online forum and a location‐based mobile networking app whose primary users were MSM. Interested individuals could either self‐refer, or be referred by community‐based organizations. All study participants gave their written consent.

### 2.3 Randomization and masking

Participants were randomized into either daily‐first arm or on‐demand‐first arm. Participants in the daily‐first arm were put on daily PrEP for 16 weeks, then on‐demand PrEP for another 16 weeks. Another arm received PrEP in a reversed regimen sequence. Arm numbers were generated centrally by using a computer and block randomization with a block size of four with a 1:1 ratio for enrollees. Participants, physicians, research support staff and investigators were not masked to the study arm allocation.

### 2.4 Procedures

All participants were instructed to take oral 300 mg TDF/200 mg FTC tablets. PrEP‐naïve participants each received one daily tablet over a period of 2 weeks prior to the 32‐week study period for assessing their tolerability before randomization. During the 16‐week daily PrEP period, they were asked to take their tablet on a daily basis irrespective of their pattern of sexual activities. During the 16‐week on‐demand PrEP period, two tablets were taken within 2–24 h before having sex, and then, if any sexual activity occurred, one tablet each was taken at 24 and 48 h after their first dose, a regimen referenced from the IPERGAY study [5]. The same as per instruction for IPERGAY, participants took a daily tablet until 2 days after their last consecutive day of sexual activities. Follow‐up visits were scheduled for Weeks 2, 8, 16, 18, 24 and 32. As the same drug was administered during the two intervention periods, no carryover effect was anticipated, therefore, no washout period was incorporated.

Either venepuncture or fingerprick was performed for the point‐of‐care tests (POCT). HBsAg was screened at the baseline and at Weeks 16 and 32, while HIV antigen/antibody were tested at every visit. STIs, including syphilis and genital, pharyngeal and rectal *Neisseria gonorrhoea* (NG) and *Chlamydia trachomatis* (CT), were tested at the baseline and every 8 weeks. Renal function was tested at all time points except at Weeks 8 and 24. Participants were informed of their POCT results at the same clinic setting, and the laboratory test results at their subsequent visit. Clinical referrals were made following the participants’ consent in the case of STI diagnosis. At the baseline, participants underwent self‐administered eligibility screening, and completed behavioural questionnaires through a web‐based password‐controlled platform. At each scheduled visit, questionnaires on sexual activities, drug adherence and tolerance were recorded. After finishing the 32 weeks’ follow‐up or upon default, an exit survey was administered. During the follow‐up period, participants completed an online diary to report any daily sexual activities and PrEP use with a recall period of up to 14 days. A visit at Week 50 was scheduled for HIV/STI testing and questionnaire completion on any sexual activities and PrEP use and preferences. POCT used included: Alere HIV Combo fourth‐generation test, SD BIOLINE syphilis 3.0, CHEMBIO DPP Syphilis Screen & Confirm (treponemal and non‐treponemal test), SD BIOLINE HBsAg and i‐STAT Creatinine test. Gonorrhoea and chlamydia were detected with nucleic acid amplification tests in urine and self‐collected pharyngeal and rectal swabs.

### 2.5 Outcomes

The primary outcome of the trial was PrEP coverage of days with CLAI, reflecting prevention‐effective adherence [17] during the two regimen periods. Secondary outcomes included (1) uptake of TDF/FTC derived from the percentage of days on PrEP and retention rate; (2) STI diagnoses through testing; and (3) regimen preferences and risk perception. Safety outcomes included adverse events graded using the Division of AIDS (DAIDS) Table for Grading the Severity of Adult and Pediatric Adverse Events [18] and change in creatinine clearance level.

### 2.6 Statistical analysis

CLAI covered by PrEP was defined by having taken two doses the day before or on the day of sex and two separate subsequent doses. A high coverage was denoted by >90% of days with CLAI covered by PrEP. Overall, the number and percentage of days on PrEP, and CLAI‐days by study arm and regimen periods were calculated. Intention‐to‐treat analysis was conducted to assess the differences in adherence, and coverage between the two arms. Generalized linear model (GLM) with binomial distribution was used to identify factors associated with high coverage. GLM with Poisson distribution was used to predict the number of days with PrEP‐covered CLAI. The linear mixed‐effect model was used to assess PrEP's effect on creatinine clearance calculated by the Cockcroft–Gault equation with random individual intercepts. Statistical significance was approximated by the Kenward–Roger method. Intra‐class correlation was measured to assess the proportion of within‐person variance over time. The proportion of participants showing interest in continuing PrEP and acceptance of either regimen upon study completion or drop‐out were measured to reflect PrEP acceptability. Factors associated with perceived HIV risk at the endpoint and potential change since enrolment were tested by chi‐squared test and the logistic regression model. R 3.6.2 was used for all statistical analyses.

## 3 RESULTS

**Figure 1:**
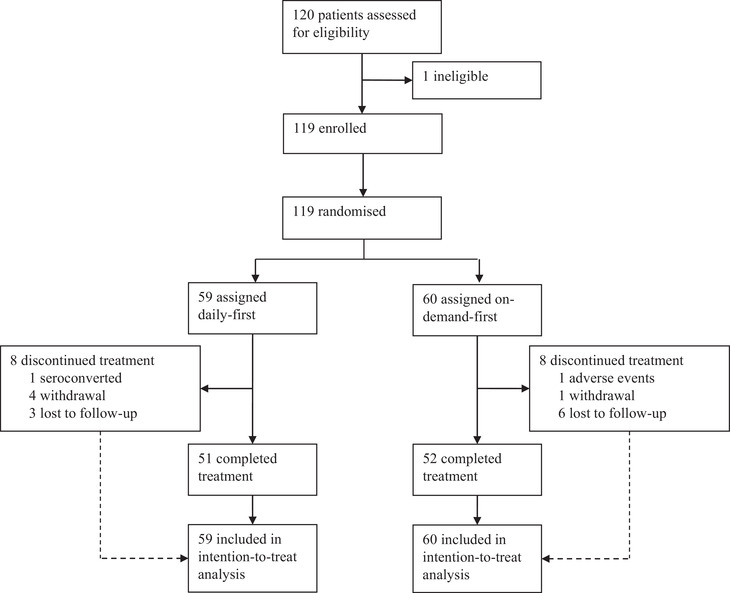
Trial profile of a pre‐exposure prophylaxis crossover study among men who have sex with men in Hong Kong.

Between 25 August 2018 and 23 March 2019, 120 MSM were screened; one person with acute HIV infection was detected and excluded. Sixty subjects were assigned to the on‐demand‐first arm, while 59 were in the daily‐first arm (Figure 1). Participants’ median age at enrolment was 30 years [interquartile range (IQR) 26–38 years] (Table 1). The majority (87%, *n* = 104) were local ethnic Chinese and the same proportion had attained at least postsecondary education level. Some 94 (86%) and 30 (28%) had group sex and sex with PLHIV prior to their enrolment, respectively. Less than half (42%, *n* = 50) were PrEP‐experienced. Weeks 16 and 32 completion rates were 94% and 87%, respectively, without inter‐arm significant difference (Table 2). One MSM in the daily‐first arm seroconverted and his infection occurred during the daily period because of poor adherence, as reported previously [19].

**Table 1:** Baseline characteristics of men who have sex with men participating in a PrEP crossover study in Hong Kong

	Overall (*N* = 119)	Daily‐first arm (*n* = 59)	On‐demand‐first arm (*n* = 60)
Demographics [*n* (%)]			
Median age (IQR), years	30 (26–38)	29 (25–38)	30 (26–40)
Local ethnic Chinese	104 (87%)	51 (86%)	53 (88%)
Postsecondary education level	104 (87%)	51 (86%)	53 (88%)
Employed fulltime	84 (71%)	40 (68%)	44 (73%)
Sexual experience [*n* (%)]			
Median age of sexual debut with men (IQR), years	19 (17–21)	19 (17–21)	19 (17–21)
Ever STI diagnosis (self‐reported)	53 (45%)	27 (46%)	26 (43%)
Ever had sex with HIV‐positive individuals	30 (25%)	18 (31%)	12 (20%)
Ever had group sex	94 (79%)	46 (78%)	48 (80%)
Ever had chemsex	63 (53%)	35 (59%)	28 (47%)
Perceived high HIV risk at baseline	28 (24%)	15 (25%)	13 (22%)
Used PrEP before	50 (42%)	24 (41%)	26 (43%)

Abbreviations: HIV, human immunodeficiency virus; IQR, interquartile range; PrEP, pre‐exposure prophylaxis; STI, sexually transmitted infection.

**Table 2:** Adherence and coverage of days with CLAI and sexual behaviour parameters collected from the diaries of men who have sex with men participating in a PrEP crossover study in Hong Kong

	Study arm		PrEP regimen	
	Daily‐first (*n* = 59)	On‐demand‐first (*n* = 60)	Daily	On‐demand
Completion rate				
Week 2	59 (100%)	59 (98%)		
Week 8	56 (95%)	57 (95%)		
Week 16	56 (95%)	56 (93%)		
Week 18	56 (95%)	55 (92%)		
Week 24	53 (90%)	53 (88%)		
Week 32	51 (86%)	52 (87%)		
Number of days on PrEP during daily period, Median (IQR)	100 (82–107)*	81 (39–105)*		
Number of days on PrEP during on‐demand period, Median (IQR)	56 (25–79)*	35 (21–57)*		
Total number of days with CLAI, Median (IQR)	10 (4–33)	15 (4–24)	5 (2–13)	7 (2–15)
Total number of days with sex with condom, Median (IQR)	1 (0–3)	1 (0–8)	0 (0–2)	0 (0–2)
Total days with PrEP taken, Median (IQR)	129 (109–161)	131 (86–171)	93 (64–106)*	45 (25–70)*
% days with PrEP, Median (IQR)	74.36 (64.29–86.39)	72.47 (54.17–84.30)	95.54 (87.85–100.00)*	53.85 (51.58–75.00)*
Total number of days with CLAI covered by PrEP, Median (IQR)	9 (3–31)	14 (2–22)	7 (3–16)	8 (2–12)
% days with CLAI covered by PrEP, Median (IQR)	92.31 (77.78–100.00)	91.88 (75.00–100.00)	100.00 (82.60–100.00)	93.30 (80.00–100.00)

Abbreviations: CLAI, condomless anal intercourse; IQR, interquartile range; PrEP, pre‐exposure prophylaxis.

Note: Categorical and continuous variables were tested by chi‐squared and Mann–Whitney U test, respectively.

* *p* < 0.05.

Overall, the median number of days with CLAI was 13 with an IQR of 4–28, 11 (IQR: 3–20) of which were covered by PrEP (Table 2). Participants in the daily‐first arm had a median of 10 (IQR: 4–33) days with CLAI (CLAI‐days), 9 (IQR: 3–31) of which were covered by PrEP. Out of a median of 15 (IQR: 4–24) CLAI‐days in the on‐demand‐first arm, 14 (IQR: 2–22) were covered. Differences in the numbers of CLAI‐days (*p* = 0.94), PrEP‐covered CLAI days (*p* = 0.97) and the proportions of days with PrEP‐covered CLAI (*p* = 0.93) were not statistically significant between the two arms. A median of 7 (IQR: 3–16) and 8 (IQR: 2–12) CLAI‐days were covered by PrEP during the daily and on‐demand periods, respectively (*p* = 0.93). The median proportion of days with PrEP‐covered CLAI was 100% (IQR: 83–100%) during the daily periods and 93% (IQR: 80–100%) during the on‐demand periods (*p* = 0.14). Sequence effect was not observed in both the number and proportion of CLAI.

The total numbers of days with sex and PrEP were not different between the two arms (Table 2). The median number of days with PrEP was 129 (IQR: 97–167), equivalent to about 73% (IQR: 59–85%) of the person‐days. The median number and percentage of days on PrEP during the daily and on‐demand periods was 93 (IQR: 64–106) days or 96% (88–100%) and 45 (IQR: 25–70) days or 54% (32–75%), respectively. The daily‐first arm had a higher median number of days on PrEP during both periods compared with the on‐demand‐first arm. Participants on the daily regimen first had more days on PrEP during the on‐demand period, while those taking the on‐demand first had less days on daily PrEP.

MSM who achieved high (>90%) coverage of CLAI‐days with PrEP were older (*p* = 0.0086), not acting as a receptive sexual role only (*p* = 0.025), had no sex partners on PrEP at the baseline (*p* = 0.031) and at Week 24 (*p* = 0.034), and had higher odds of seeking sex partners at Week 24 (*p* = 0.044) (Table 3). After adjusting for the number of CLAI‐days, participants younger than 30 years old (*p* = 0.022) and whose sex partners were on PrEP (*p* = 0.0062) had lower odds of achieving high coverage. The total number of days with PrEP‐covered CLAI was, after adjusting for the total CLAI‐days, age and study arm, associated with completion of 32 weeks follow‐up (*p*<0.0001), high perceived risk of HIV infection at the baseline (*p* = 0.0045), inclination to have CLAI as an insertive role at the baseline (*p*<0.0001) and engagement in chemsex (*p*<0.0001) within 6 months prior to study participation (Table 4).

**Table 3:** Comparison between MSM with high (>90%) and low (≤90%) proportion of days with PrEP‐covered CLAI in a crossover study in Hong Kong

	Coverage >90% (*n* = 63)	Coverage ≤90% (*n* = 48)	OR (95% CI)	*p*‐value	aOR (95% CI)	*p*‐value
Age under 30 years old	25 (40%)	32 (67%)	0.33 (0.15–0.72)	0.0086	0.36 (0.15–0.85)	0.022
Receptive sexual role	15 (24%)	22 (46%)	0.37 (0.16–0.83)	0.025	–	–
Sex partner on PrEP at baseline	15 (25%)	22 (47%)	0.38 (0.17–0.86)	0.032	0.28 (0.11–0.68)	0.0062
Sought sex partners at Week 24	51 (88%)	30 (70%)	3.16 (1.13–8.79)	0.044	–	–
Median number of CLAI during study period (IQR)	10 (4–26)	18 (7–34)	1254^a^	0.124	1.02 (1.00–1.04)	0.122

Abbreviations: aOR, adjusted odds ratio; CI, confidence interval; CLAI, condomless anal intercourse; IQR, interquartile range; MSM, men who have sex with men; OR, odds ratio; PrEP, pre‐exposure prophylaxis.

Note: Univariable analysis was performed by chi‐squared and Mann–Whitney U test, while generalized linear model was performed to identify significant predictors.

^a^ *W*‐value from the Mann–Whitney U test.

**Table 4:** Predictors of the total number of days with PrEP‐covered CLAI among men who have sex with men participating in a PrEP crossover study in Hong Kong

	aOR (95% CI)	*p*‐value
Number of days with CLAI during study period	1.03 (1.02–1.03)	<0.0001
Study arm	0.84 (0.77–0.92)	0.00013
Age under 30 years old	1.10 (0.99–1.22)	0.081
Completed Week 32 follow‐up	1.64 (1.33–2.05)	<0.0001
Postsecondary education level	0.81 (0.72–0.91)	0.00052
High perceived risk of HIV infection at baseline	1.14 (1.042–1.26)	0.0045
Inclination to have CLAI as insertive role at baseline	1.78 (1.57–2.02)	<0.0001
Engagement in chemsex before enrolment	1.35 (1.24–1.48)	<0.0001

Abbreviations: aOR, adjusted odds ratio; CI, confidence interval; CLAI, condomless anal intercourse; PrEP, pre‐exposure prophylaxis.

Note: GLM with Poisson distribution model was employed for predicting the number of PrEP‐covered CLAI.

Almost half (47%, 53/113) had at least one incident STI during the follow‐up period, with an incidence rate of 87.46 per 100 person‐years (py) (Table 5). About 12% (13/113) tested positive for syphilis during their follow‐up, with an incidence rate of 17.74/100 py. Totally, 48 incident NG infections at various sites were detected in the 32 participants (*n* = 112). The incidence rate of all‐site NG and CT was 45.95/100 py and 50.29/100 py, respectively. Participants in the daily‐first arm had similar odds of having incident STI as those in another arm (*p* = 0.072).

**Table 5:** STI incidence by anatomical site and type among men who have sex with men participating in a PrEP crossover study in Hong Kong

	*n*/*N* (%)	Incidence (per 100 py)
Any STI	53/113 (47%)	87.46
Syphilis	13/113 (12%)	17.74
Any‐site NG	32/112 (29%)	45.95
Pharyngeal NG	23/103 (22%)	41.37
Rectal NG	22/100 (22%)	40.93
Genital NG	3/111 (3%)	4.57
Any‐site CT	35/113 (31%)	50.29
Rectal CT	21/93 (23%)	43.35
Pharyngeal CT	6/111 (6%)	9.34
Genital CT	15/110 (14%)	24.01

Abbreviations: CLAI, condomless anal intercourse; CT, *Chlamydia trachomatis*; NG, *Neisseria gonorrhea*; PrEP, pre‐exposure prophylaxis; py, person‐years; STI, sexually transmitted infections.

Over one‐third (36%, *n* = 43) reported different grades of adverse events, of which the commonest were diarrhoea, headache, nausea and dizziness (Table 6). Three quarters (77%, *n* = 33) reported adverse events with daily regimen only, with all but one completing all follow‐up visits. Nine out of 37 participants with adverse events during the daily regimen had persistent symptoms throughout the entire 16 weeks. Five of the 10 participants reporting adverse events during the on‐demand period attributed their symptoms to the loading double‐dose. All but one reported grade 1 adverse events. The participant who reported grade 2 nausea, depression and sleep disturbance was taking on‐demand PrEP until Week 16, during which he reported 94 days of PrEP use before withdrawal. Overall, the reported adverse events lasted for a median of 14 days (IQR: 4–63 days). The change of creatinine clearance over time was –0.39 ml/min (95% CI: –0.49 to –0.28, *p*<0.0001) per week from an intercept of 120.12 (95% CI: 115.48–124.75, *p*<0.0001), with no difference between the two arms. A higher proportion (74.11%) of variance was attributable to between‐person variation, while only 25.89% was attributable to within‐person variation.

**Table 6:** Adverse events reported by men who have sex with men participating in a crossover study in Hong Kong by PrEP regimen (*n* = 43)

	Daily^a^	On‐demand^a^
Diarrhoea	7 (16%)	4 (9%)
Nausea	4 (9%)	3 (7%)^b^
Dyspepsia	7 (16%)	2 (5%)
Palpitation	1 (2%)	0 (0%)
Lethargy	6 (14%)	0 (0%)
Dizziness	6 (14%)	2 (5%)
Dry skin	1 (2%)	0 (0%)
Headache	8 (19%)	0 (0%)
Depression	1 (2%)	1 (2%)^b^
Sleep disturbance	4 (9%)	1 (2%)^b^
Joint pain	1 (2%)	0 (0%)
Number of participants reported adverse events (*N* = 119)	37 (31%)	10 (8%)

Abbreviation: PrEP, pre‐exposure prophylaxis.

^a^ Grades are determined by the Division of AIDS (DAIDS) Table for Grading the Severity of Adult and Pediatric Adverse Events.

^b^ Including one grade 2 adverse event.

Upon completion of or withdrawal from the study, almost all (96%, 105/109) indicated their intention to continue PrEP for HIV prevention. Sixteen (15%) participants accepted both daily and on‐demand PrEP, while 44 (40%) and 43 (39%) showed preference only for daily and on‐demand PrEP, respectively. More participants had lowered perceived risk of HIV infection compared with the baseline (39% vs. 17%). The 18 (17%) participants who considered themselves as having high risk of HIV infection at the endpoint were more likely to have sex partners on PrEP at the baseline (*p* = 0.012), report STI diagnosis at Week 16 (*p* = 0.026) and have an emotionally attached partner at Week 24 (*p* = 0.016) (Table 7). Multiple logistic regression showed that having sex partners on PrEP at the baseline (*p* = 0.024) and reporting STI diagnosis at Week 16 (*p* = 0.017) increased one's odds of perceiving high HIV risk at the endpoint.

**Table 7:** Characteristics of men who have sex with men participants by perceived HIV risk at the endpoint in a PrEP crossover study in Hong Kong

	Perceived risk always high (*n* = 18)	Perceived risk not always high (*n* = 91)	OR (95% CI)	*p*‐value	aOR (95% CI)	*p*‐value
Age of sexual debut ≤21 years	10 (59%)	76 (84%)	0.26 (0.09–0.81)	0.035	0.25 (0.07–0.90)	0.033
Sex partners at baseline were on PrEP	11 (61%)	24 (27%)	4.19 (1.46–12.06)	0.012	3.82 (1.21–12.73)	0.024
Reported STI diagnosis at Week 16	8 (44%)	15 (17%)	3.84 (1.30–11.35)	0.026	4.70 (1.32–17.68)	0.017
Had emotionally attached partners at Week 24	3 (17%)	1 (1%)	16.80 (1.64–172.48)	0.016	–	–

Abbreviations: aOR, adjusted odds ratio; CI, confidence interval; HIV, human immunodeficiency virus; OR, odds ratio; PrEP, pre‐exposure prophylaxis; STI, sexually transmitted infection.

Note: Univariable and multivariable analysis was performed by chi‐squared test and logistic regression model, respectively.

## 4 DISCUSSION

We have reported here the outcomes of daily versus on‐demand PrEP in MSM. In the study, we chose the coverage of days with CLAI as the primary measure, the definition of which was adapted from the IPERGAY regimen [5]. Adherence is the conventional measure in monitoring PrEP uptake, but its varied definition between daily and on‐demand regimen causes problems in making comparisons. Reflecting high prevention‐effective adherence, our results showed an over 90% coverage of days with CLAI by either regimen, notwithstanding the almost doubled doses taken during the daily periods. All CLAI events were covered in at least half of the MSM, and over half of the MSM on daily PrEP could achieve a high, over 95%, adherence. While we did not observe any sequence effects relating to the frequency of CLAI, the initiation of one regimen might have affected the pattern of PrEP use by a subsequent regimen. A higher coverage among those having no sex partners on PrEP could be a result of their higher sexual health consciousness and concern over sex partners’ HIV protection practices [20]. Their perceived and actual risk like chemsex also resulted in their higher coverage. The lower coverage among younger MSM may require additional interventions like the use of mobile apps in some research [21]. In our study, only one MSM seroconverted while on daily PrEP, as a result of suboptimal adherence, giving an incidence of 1.40/100 py (95% CI: 0.20–9.94). Our findings echoed the phenomenon of the rarity of PrEP failure and the importance of ensuring adequate coverage of CLAI‐days irrespective of regimen.

In implementing PrEP, reduced condom use has always been a concern. Actual bacterial STI risk was high as almost half had acquired at least one confirmed infection corresponding with an incidence of 87.46 per 100 py. A considerable number of incident rectal infections, including NG and CT, were found, similar to those reported in a Thai study [22], signifying the importance of testing multiple anatomical sites for STI. Separately, tolerability is a yardstick for the assessment of acceptability. Our 36% adverse events rate was higher than another study [6], but both showed the predominance of mild gastrointestinal symptoms. In our study, almost all MSM with adverse effects suffered transient discomfort lasting for no more than 2 weeks and all except one participant had continued taking PrEP throughout the study period. Similar to a study in San Francisco, some participants reported adverse events triggered by the loading dose during on‐demand PrEP [23]. While concern for potential adverse events of daily PrEP has often been cited as a reason for opting for on‐demand regimens [24], results from clinical studies do not support the strategy.

Our 32‐week retention rate was 87%, indicating the high acceptance of PrEP delivered in a research clinic setting. A similar proportion favoured daily and on‐demand PrEP, which showed that MSM with distinct sexual behaviour patterns [8] and health needs may have different preferences of PrEP regimens, notably, the ability to predict time of sexual activities and perceived protection effect [25]. Having both regimens in place can allow for the flexible use of either regimen for MSM at different time periods. Putting on daily PrEP at initiation served not just to assess its tolerability but the purpose of reinforcing the importance of adherence, the effect of the latter could last after switching to an on‐demand regimen. In addition to eliciting MSM's acceptance of PrEP, their wish to remain on PrEP in the following years reflected their satisfaction with the experiences in meeting sexual health needs.

We acknowledge that our study carries some limitations. Extrapolation of the study results should be cautioned because societal norms could be different elsewhere and the participants’ high acceptance of the study setting may be affected by the lack of affordable alternative access for PrEP in Hong Kong. Sexual behaviours and diary data were self‐reported, which may have been defected by social desirability and recall bias. The short recall period (up to 3 months for behavioural surveys and 14 days for diaries) could minimize recall bias and the use of computer‐assisted self‐administered questionnaires could have facilitated data entry in confidence. Also, the Hawthorne effect might have contributed to the change of sexual behaviours. Our measurement of the coverage of days with CLAI by PrEP could have been overestimated as the exact dose time was not taken into account, especially for the loading dose of the on‐demand regimen prior to sex. Our definition of coverage was stricter than that of IPERGAY which required that only one dose was required to have been taken within 24 h before, plus another within 48 h after sex. Although four doses per week gave a considerable protective effect [26], such a definition would ignore the effects of pre‐ and post‐coital doses, and was not adopted in the study so as not to over‐estimate the PrEP's coverage. Future studies could incorporate additional tests or tools, such as DBS [19] or smart caps, to validate the accuracy of diary data. In the assessment of STI, their inherent window periods and our referral of patients out for treatment could have affected the incidence endpoints. As we only analysed laboratory test results during the study periods, the STI history could have been incomplete though overestimation might still have occurred as recently treated infections could have been diagnosed as incident cases. The 2‐week lead‐in period with daily TDF/FTC might have caused asymmetry in the two arms. We did not, however, include data during the lead‐in period for assessing adherence and coverage. The short follow‐up periods for each regimen may also have limited the observation on one's tolerance, adherence and CLAI on a longer term. With a crossover study design, we were able to adjust for inter‐person variability when comparing one's acceptance of and adherence to different PrEP regimens, but the controlled environment may have limited its clinical application in practice. This prompts a future direction for implementation and research on a flexible PrEP regimen [27].

## 5 CONCLUSIONS

Both daily and on‐demand PrEP provided similar coverage of sex acts, which were tolerated and accepted by MSM. The high STI incidence irrespective of regimen highlighted the importance of regular multi‐site STI screening for PrEP‐using MSM. Considering MSM's individual preference and pattern of sexual behaviours, flexible PrEP regimen with switching could be a strategic approach for effective programme implementation.

## CONFLICT OF INTEREST

SS has served as a member of the Advisory Boards of Merck, GSK and Gilead Sciences, and received grants and drug sponsorship from Gilead Sciences. GCY has served as an advisory committee member for Gilead, Merck, Sanofi Pasteur and GSK, speaker for Merck and Gilead, and has received research grants from Gilead, Merck, Janssen and GSK.

## AUTHOR CONTRIBUTIONS

All authors contributed to the design of the study. SS, GCY and KCK were responsible for the day‐to‐day running of the research clinic. TH, NS and DPC supported the trial operation. TTN, SS and TH contributed to the analysis plan. TH, SS and TTN analysed the data. TH and SS wrote the first draft of the article. All authors revised the draft and approved the final version.

## ROLE OF THE FUNDING SOURCE

The funder of the study had no role in the study design, data collection, data analysis, data interpretation or writing of the report. The corresponding author had full access to all of the data in the study and had final responsibility for the decision to submit for publication.

## References

[1] Grant RM, Lama JR, Anderson PL, McMahan V, Liu AY, Vargas L, et al. Preexposure chemoprophylaxis for HIV prevention in MSM. N Engl J Med. 2010;363(27):2587–99. 10.1056/NEJMoa1011205. 21091279 PMC3079639

[2] Fonner VA, Dalglish SL, Kennedy CE, Baggaley R, O'Reilly KR, Koechlin FM, et al. Effectiveness and safety of oral HIV preexposure prophylaxis for all populations. AIDS. 2016;30:1973–83. 10.1097/QAD.0000000000001145. 27149090 PMC4949005

[3] Department of HIV/AIDS . Consolidated guidelines on HIV prevention, diagnosis, treatment and care for key populations. Switzerland: WHO Press; 2016. 27559558

[4] Centers for Disease Control and Prevention Preexposure prophylaxis for the prevention of HIV infection in the US—2014: a clinical practice guideline. 2014. http://www.cdc.gov/hiv/pdf/prepguidelines2014.pdf

[5] Molina JM, Capitant C, Spire B, Pialoux G, Cotte L, Charreau I, et al. On‐demand preexposure prophylaxis in men at high risk for HIV‐1 infection. N Engl J Med. 2015;373(23):2237–46. 10.1056/NEJMoa1506273. 26624850

[6] Molina JM, Charreau I, Spire B, Cotte L, Chas J, Capitant C, et al. Efficacy, safety, and effect on sexual behaviour of on‐demand pre‐exposure prophylaxis for HIV in men who have sex with men: an observational cohort study. Lancet HIV. 2017;4(9):e402–10. 10.1016/S2352-3018(17)30089-9. 28747274

[7] Siguier M, Mera R, Pialoux G, Ohayon M, Cotte L, Valin N, et al. First year of pre‐exposure prophylaxis implementation in France with daily or on‐demand tenofovir disoproxil fumarate/emtricitabine. J Antimicrob Chemother. 2019;74(9):2752–58. 10.1093/jac/dkz220. 31219561

[8] Hoornenborg E, Coyer L, Achterbergh RCA, Matser A, Schim van der Loeff MF, Boyd A, et al. Sexual behaviour and incidence of HIV and sexually transmitted infections among men who have sex with men using daily and event‐driven pre‐exposure prophylaxis in AMPrEP: 2 year results from a demonstration study. Lancet HIV. 2019;6(7):e447–55. 10.1016/S2352-3018(19)30136-5. 31178284

[9] Vuylsteke B, Reyniers T, De Baetselier I, Nöstlinger C, Crucitti T, Buyze J, et al. Daily and event‐driven pre‐exposure prophylaxis for men who have sex with men in Belgium: results of a prospective cohort measuring adherence, sexual behaviour and STI incidence. J Int AIDS Soc. 2019;22(10):e25407. 10.1002/jia2.25407. 31663257 PMC6819896

[10] Wu D, Tao H, Dai J, Liang H, Huang A, Zhong X. Study on pre‐exposure prophylaxis regimens among men who have sex with men: a prospective cohort study. Int J Environ Res Public Health. 2019;16(24):4996. 10.3390/ijerph16244996. PMC6950352 31818006

[11] Lee SS, Kwan TH, Wong NS, Lee KCK, Chan DPC, Lam TTN, et al. Piloting a partially self‐financed mode of human immunodeficiency virus pre‐exposure prophylaxis delivery for men who have sex with men in Hong Kong. Hong Kong Med J. 2019;25(5):382–91. doi: 10.12809/hkmj198030. 31619578 10.12809/hkmj198030

[12] Grant RM, Mannheimer S, Hughes JP, Hirsch‐Moverman Y, Loquere A, Chitwarakorn A, et al. Daily and nondaily oral preexposure prophylaxis in men and transgender women who have sex with men: the HIV Prevention Trials Network 067/ADAPT Study. Clin Infect Dis. 2018;66(11):1712–21. 10.1093/cid/cix1086. 29420695 PMC5961078

[13] Bekker LG, Roux S, Sebastien E, Yola N, Amico KR, Hughes JP, et al. Daily and non‐daily pre‐exposure prophylaxis in African women (HPTN 067/ADAPT Cape Town Trial): a randomised, open‐label, phase 2 trial. Lancet HIV. 2018;5(2):e68–78. 10.1016/S2352-3018(17)30156-X. 28986029 PMC6107917

[14] Cornelisse VJ, Lal L, Price B, Ryan KE, Bell C, Owen L, et al. Interest in switching to on‐demand HIV pre‐exposure prophylaxis (PrEP) among Australian users of daily PrEP: an online survey. Open Forum Infect Dis. 2019;6(7):ofz287. 10.1093/ofid/ofz287. 31304192 PMC6612821

[15] Zimmermann HM, Eekman SW, Achterbergh RC, Schim van der Loeff MF, Prins M, de Vries HJ, et al. Motives for choosing, switching and stopping daily or event‐driven pre‐exposure prophylaxis – a qualitative analysis. J Int AIDS Soc. 2019;22(10):e25389. 10.1002/jia2.25389. 31612621 PMC6791997

[16] Koppe U, Marcus U, Albrecht S, Jansen K, Jessen H, Gunsenheimer‐Bartmeyer B, et al. Factors associated with the informal use of HIV pre‐exposure prophylaxis in Germany: a cross‐sectional study. J Int AIDS Soc. 2019;22(10):e25395. 10.1002/jia2.25395. 31583823 PMC6776824

[17] Haberer JE, Bangsberg DR, Baeten JM, Curran K, Koechlin F, Amico KR, et al. Defining success with HIV pre‐exposure prophylaxis: a prevention‐effective adherence paradigm. AIDS. 2015;29(11):1277–85. 10.1097/QAD.0000000000000647. 26103095 PMC4480436

[18] U.S. Department of Health and Human Services, National Institutes of Health, National Institute of Allergy and Infectious Diseases, Division of AIDS Division of AIDS (DAIDS) Table for Grading the Severity of Adult and Pediatric Adverse Events, Corrected Version 2.1. 2017. https://rsc.niaid.nih.gov/sites/default/files/daidsgradingcorrectedv21.pdf

[19] Lee SS, Anderson PL, Kwan TH, Lui GCY, Chan DPC, Wong NS, et al. Failure of pre‐exposure prophylaxis with daily tenofovir/emtricitabine and the scenario of delayed HIV seroconversion. Int J Infect Dis. 2020;94:41–3. 10.1016/j.ijid.2020.03.014. 32173577

[20] Kwan TH, Lee SS. Bridging awareness and acceptance of pre‐exposure prophylaxis among men who have sex with men and the need for targeting chemsex and HIV testing: cross‐sectional survey. JMIR Public Health Surveill. 2019;5(3):e13083. 10.2196/13083. 31271148 PMC6636239

[21] Finkenflügel RNN, Hoornenborg E, Achterbergh RCA, Marra E, Davidovich U, de Vries HJC, et al. A mobile application to collect daily data on preexposure prophylaxis adherence and sexual behavior among men who have sex with men: use over time and comparability with conventional data collection. Sex Transm Dis. 2019;46(6):400–6. 10.1097/OLQ.0000000000000999. 30882717 PMC6553988

[22] Hiransuthikul A, Sungsing T, Jantarapakde J, Trachunthong D, Mills S, Vannakit R, et al. Correlations of chlamydia and gonorrhoea among pharyngeal, rectal and urethral sites among Thai men who have sex with men: multicentre community‐led test and treat cohort in Thailand. BMJ Open. 2019;9(6):e028162. 10.1136/bmjopen-2018-028162. PMC6609041 31253622

[23] Broussard J, Bena J, Crouch PC, Taylor B, Chavez M, Grant RM. PrEP 2‐1‐1 education increases PrEP uptake and preserves effective PrEP coverage in a large nurse‐led community‐based sexual health clinic in San Francisco. 23rd International AIDS Conference, abstract OAC0503. 2020.

[24] Hojilla JC, Marcus JL, Silverberg MJ, Hare CB, Herbers R, Hurley L, et al. Early adopters of event‐driven human immunodeficiency virus pre‐exposure prophylaxis in a large healthcare system in San Francisco. Clin Infect Dis. 2020;71(10):2710–2. 10.1093/cid/ciaa474. 32494806 PMC7744988

[25] Patel RR, Crane JS, López J, Chan PA, Liu AY, Tooba R, et al. Pre‐exposure prophylaxis for HIV prevention preferences among young adult African American men who have sex with men. PLoS One. 2018;13(12):e0209484. 10.1371/journal.pone.0209484. 30592756 PMC6310267

[26] Anderson PL, Glidden DV, Liu A, Buchbinder S, Lama JR, Guanira JV, et al. Emtricitabine‐tenofovir concentrations and pre‐exposure prophylaxis efficacy in men who have sex with men. Sci Transl Med. 2012;4(151):151ra125. 10.1126/scitranslmed.3004006. PMC3721979 22972843

[27] Wu HJ, Ku SW, Li CW, Ko NY, Yu T, Chung AC, et al. Factors associated with preferred pre‐exposure prophylaxis dosing regimen among men who have sex with men in real‐world settings: a mixed‐effect model analysis. AIDS Behav. 2021;25(1):249–58. 10.1007/s10461-020-02964-5. 32643021

